# Reducing the workload of medical diagnosis through artificial intelligence: A narrative review

**DOI:** 10.1097/MD.0000000000041470

**Published:** 2025-02-07

**Authors:** Jinseo Jeong, Sohyun Kim, Lian Pan, Daye Hwang, Dongseop Kim, Jeongwon Choi, Yeongkyo Kwon, Pyeongro Yi, Jisoo Jeong, Seok-Ju Yoo

**Affiliations:** a College of Medicine, Dongguk University, Gyeongju-si, Republic of Korea; b Department of Preventive Medicine, College of Medicine, Dongguk University, Gyeongju-si, Republic of Korea.

**Keywords:** artificial intelligence, diagnosis, efficiency, workload

## Abstract

Artificial intelligence (AI) has revolutionized medical diagnostics by enhancing efficiency, improving accuracy, and reducing variability. By alleviating the workload of medical staff, AI addresses challenges such as increasing diagnostic demands, workforce shortages, and reliance on subjective interpretation. This review examines the role of AI in reducing diagnostic workload and enhancing efficiency across medical fields from January 2019 to February 2024, identifying limitations and areas for improvement. A comprehensive PubMed search using the keywords “artificial intelligence” or “AI,” “efficiency” or “workload,” and “patient” or “clinical” identified 2587 articles, of which 51 were reviewed. These studies analyzed the impact of AI on radiology, pathology, and other specialties, focusing on efficiency, accuracy, and workload reduction. The final 51 articles were categorized into 4 groups based on diagnostic efficiency, where category A included studies with supporting material provided, category B consisted of those with reduced data volume, category C focused on independent AI diagnosis, and category D included studies that reported data reduction without changes in diagnostic time. In radiology and pathology, which require skilled techniques and large-scale data processing, AI improved accuracy and reduced diagnostic time by approximately 90% or more. Radiology, in particular, showed a high proportion of category C studies, as digitized data and standardized protocols facilitated independent AI diagnoses. AI has significant potential to optimize workload management, improve diagnostic efficiency, and enhance accuracy. However, challenges remain in standardizing applications and addressing ethical concerns. Integrating AI into healthcare workforce planning is essential for fostering collaboration between technology and clinicians, ultimately improving patient care.

## 1. Introduction

The diagnostic process serves as the foundation for medical practice, guiding clinical decisions and shaping patient treatment outcomes. A well-timed and accurate diagnosis reduces the risks associated with delayed or inaccurate assessments and guarantees effective treatment. However, conventional diagnostic processes face several challenges: the increasing number of patients and limited manpower of doctors usually lengthen the diagnosis time and increase the workload of medical staff. In addition, dependence on subjective interpretation can lead to differences in diagnostic accuracy. These challenges emphasize the critical need for innovations to improve the efficiency and accuracy of medical diagnostics.

Artificial intelligence (AI) is a revolutionary technology that can distinguish between these tasks. AI shows the revolutionary potential of the analytical process for analyzing patterns in vast amounts of data. This potential has become increasingly evident during the coronavirus disease 2019 (COVID-19) pandemic, thereby disclosing the limitations of traditional diagnostic methods and accelerating the adoption of AI technologies.

AI is currently being utilized in various fields of healthcare, such as decreasing the amount of time between patient screening and final diagnosis, increasing accuracy, lowering hospitalization expenses, and reducing workload. IDx-DR, one example of an AI that detects diabetic retinopathy, has received Food and Drug Administration approval and is being used at the Endocrinology Center at UI Health Care–Iowa River Landing in Coralville.^[1–3]^

Previous studies primarily evaluated the role of AI in specific specialties. However, comprehensive analyses of its impact on reducing clinical workload across diagnostic fields are limited. Moreover, accurately predicting future healthcare workforce dynamics remains challenging, particularly because AI integration may shift the demand for medical staff and reshape workforce planning.^[4,5]^ We comprehensively analyzed the trends in AI applications in diagnostic methods from 2019 to 2024. Unlike previous studies that focused on improving diagnostic accuracy and reducing false positives, we aimed to highlight how AI enhances efficiency by reducing the time and data volume of clinicians during the diagnostic process.

By addressing this research gap, this study provides valuable insight into how AI can transform future healthcare diagnostics. Through this narrative review, we provide actionable insights into the role that AI will play in shaping the future of healthcare diagnostics, addressing current challenges, and redefining workforce dynamics in an evolving clinical landscape.

## 2. Subjects and methods of study

### 2.1. Literature search strategy

We conducted a literature search of PubMed from January 2019 to February 2024 for studies that assessed AI models that improved the diagnostic efficiency of clinicians. Our search included the following combination of keywords and MeSH terms: (“artificial intelligence” OR “AI”) AND (“efficiency” OR “workload”) AND (“patient” OR “clinical”).

The initial literature search yielded 2587 records for screening.

### 2.2. Inclusion criteria

Six independent reviewers screened titles and abstracts to identify potential articles. In cases of disagreement, 3 other reviewers were consulted for consensus: the use of AI models as a diagnostic tool; study with human patients; and published in English.

In all, 1192 articles met the inclusion criteria, of which 840 were retrieved in full-text review.

### 2.3. Eligibility assessment

Six researchers applied the following exclusion criteria. In case of disagreement, 3 other reviewers were consulted for consensus.

#### 2.3.1. Inappropriate article type (n = 380)

Original research articles were exclusively analyzed to ensure that the findings were based on primary data sources, avoiding potential biases or duplicate data extraction from previous review articles.

#### 2.3.2. No comparison made between human and AI (n = 101)

Studies that did not directly compare the AI model or an AI-aided clinician with a human clinician (single or team) were excluded because of the ambiguity in showing the role of AI. For instance, studies comparing a new AI model with other AI models and not clinicians were ruled out.

#### 2.3.3. Unable to extract comparative data (n = 58)

Studies without any specific data referring to the comparison between AI and clinician performance were also excluded.

#### 2.3.4. Other reasons (n = 350)

Of the 840 full-text articles that underwent thorough assessment, many were proven inadequate for review for various reasons. For example, studies focusing on clinicians’ feedback after using AI were excluded; studies presenting the possibility of AI adoption in specific areas without actual conduction of an experiment were excluded.

Finally, 51 articles were included and cited in this review.

### 2.4. Quality assessment

The quality of the included studies was assessed using appropriate tools based on study design. The Newcastle-Ottawa Scale was applied in cohort and case-control studies, and the Joanna Briggs Institute Checklist was applied in experimental and evaluation studies. In addition, the Quality Assessment of Diagnostic Accuracy Studies was applied as a quality assessment tool for diagnostic studies, and the Risk of Bias in Nonrandomized Studies of Interventions (ROBINS-I) was applied as a quality assessment tool for nonrandomized intervention studies.

**Figure 1.**
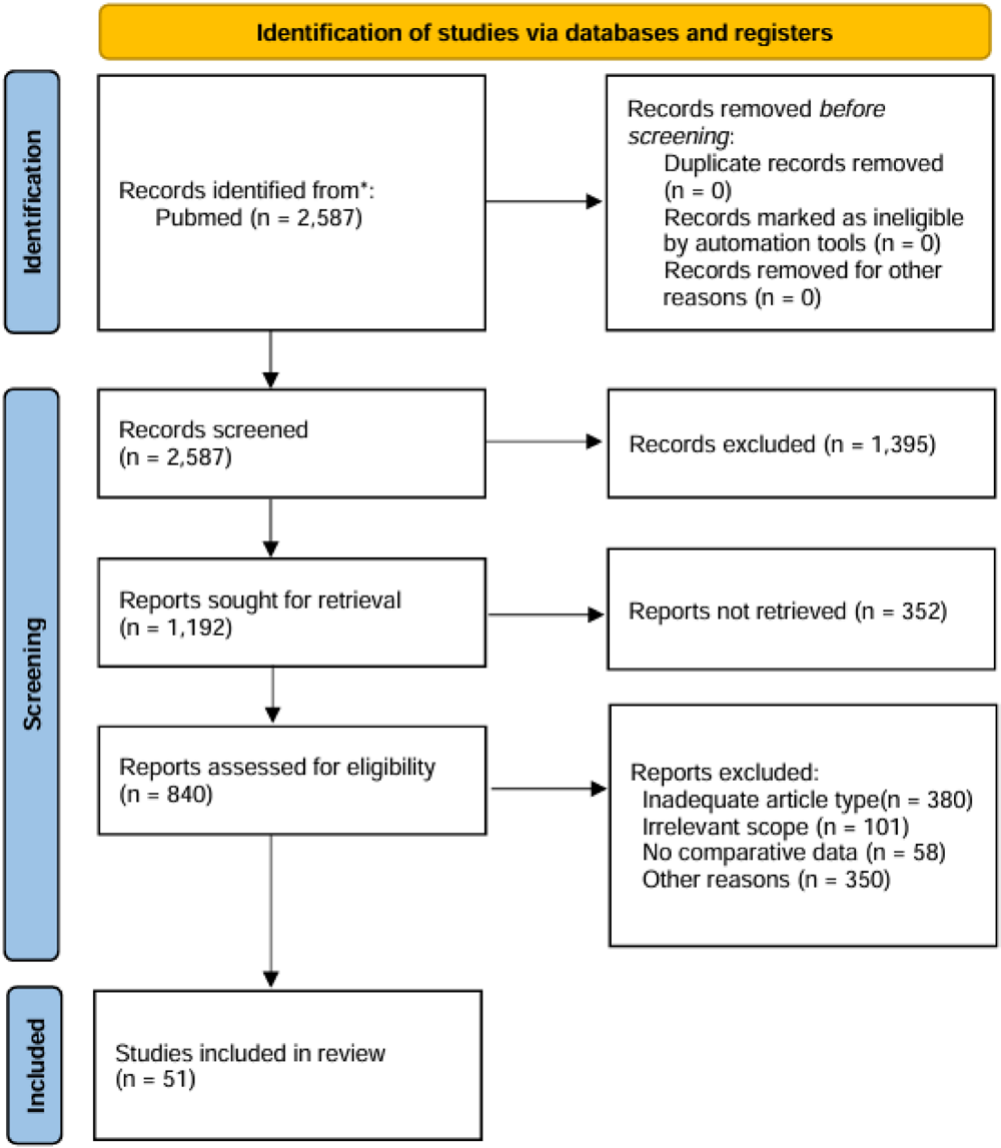
Flowchart for retrieving study records on artificial intelligence enhancing clinicians’ efficiency based on a PubMed keyword search. “n” indicates the number of articles.

A flow diagram of the literature search strategy is shown in Figure 1. Although this review is not systematic, we partially implemented the Preferred Reporting Items for Systematic Reviews and Meta-Analyses 2020 flow diagram for increased transparency.

## 3. Results

The final 51 articles were reviewed and classified into 4 categories, as presented in table. The classification was based on the quantifying factors of diagnostic efficiency and detailed diagnostic mechanisms. The factors of efficiency presentation include changes in diagnostic time and volume of data requiring review during the diagnostic process. These 2 factors were selected because their changes directly indicate changes in the diagnostic efficiency.

Changes in diagnostic time were further divided into 3 detailed subcategories:

### 3.1. Provision of supporting material for clinicians’ decision-making (A)

AI provides supportive materials to clinicians, such as annotated images indicating suspected lesion sites, to support them in making final diagnoses. In these cases, AI does not reach independent diagnostic capability but helps enhance diagnostic efficiency and points toward a collaborative model between clinicians and AI.

### 3.2. Reduction in the volume of data requiring review during diagnosis (B)

This involves AI filtering out images that do not require further review and presenting only those images essential for clinicians to examine. AI takes over the roles of tiring and repetitive tasks. This reduces clinicians’ workload and enables them to focus on more critical diagnostic tasks.

### 3.3. Independent diagnosis by AI (C)

This would involve the AI completing the diagnostic process independently, with no further need for intervention by clinicians. This would ensure maximum efficiency in diagnosis and is thus very promising regarding AI for workforce shortages.

If the study only reported a reduction in the volume of data, clinicians needed to review, without measuring, changes in time required for diagnosis; it was categorized as “volume reduction of data requiring review without time measurement” (D).

The data were classified according to the following criteria: 56.86%, 5.88%, 25.49%, and 11.76%, respectively. As a result of classifying in detail by the classification criteria according to the American Board of Medical Specialties, it was found that internal medicine (cardiovascular medicine) 3.92%, internal medicine (hematology) 5.88%, gastroenterology 7.84%, neurological surgery 1.96%, neurology 1.96%, nuclear medicine 1.96%, ophthalmology 3.92%, pathology 15.699%, radiology 54.90%, and urology 1.96%. A detailed breakdown of all the included studies is provided in Tables 1 and 2.

**Table 1:** AI Applications and Diagnostic Time Outcomes Across Medical Specialties*

Lead author	Year	Specialty	Disease	Outcome	Sample size	Result	ABCD classification†
Zheng^[6]^	2023	Radiology	Breast cancer	Diagnosis of single-mass breast lesions on contrast-enhanced mammography	1912	99.67% reduction in diagnosis time	A
Yacoub^[7]^	2022	Radiology	Pulmonary disease	Automated detection and segmentation of lung and cardiac findings on chest CT	390	22.1% reduction in diagnosis time	A
Raya-Povedano^[8]^	2021	Radiology	Breast cancer	Breast cancer screening on DBT	15,987	72.2% reduction in diagnosis time	B
Li^[9]^	2023	Radiology	Fresh rib fracture	Fresh rib fracture detection and positioning	2319	95% reduction in diagnosis time	C
Shi^[10]^	2020	Radiology	Intracranial aneurysm	Detection of intracranial aneurysm	374	Radiologists: 39.53% reduction in diagnosis time Neurosurgeons: 18.75% reduction in diagnosis time	C
Ni^[11]^	2020	Radiology	Pulmonary disease	Detection of lung lesions from COVID-19 patients	96	52.82% reduction in diagnosis time	A
Ahn^[12]^	2022	Radiology	Pulmonary disease	Improving reader performance on chest radiographs	497	10% reduction in diagnosis time	A
Bolocan^[13]^	2023	Radiology	Renal cell carcinoma	Distinction between malignant and benign tissues on CT, determination of the subtype if malignant.	1073	97.14% reduction in diagnosis time	A
Booz^[14]^	2020	Radiology	BA assessment	Assessment of pediatric BA in radiographs	514	Mean evaluation times 86.9% reduction in diagnosis time Mean reading times 88.5% reduction in diagnosis time	C
Buchlak^[15]^	2023	Radiology	Intracranial disease	Detection of intracranial pathology on noncontrast CT of the brain	2848	11.23% reduction in diagnosis time	A
Cui^[16]^	2020	Radiology	Pulmonary nodules	Detection of pulmonary nodules on CT	582	95.32% reduction in diagnosis time	C
Faghani^[17]^	2024	Radiology	Gout	Identification and classification of gouty crystals using dual energy CT	30	3.68%, 4.45% reduction in diagnosis time	A
Govindarajan^[18]^	2022	Radiology	Thoracic disease	Assessing the quality of qXR to predict CXR	65,604	40.63% reduction in diagnosis time	A
Guermazi^[19]^	2021	Radiology	Fractures	Assistance in radiographic fracture recognition for physicians	5760	11% reduction in diagnosis time	A
Hatipoglu^[20]^	2022	Radiology	Myocardial disease	Biventricular volumetric analysis on CMR	300	97.63% reduction in diagnosis time	C
Huo^[21]^	2023	Radiology	Lung cancer	Automatic detection of bone metastases in lung cancer on CT	38	61.57% reduction in diagnosis time	A
Liu^[22]^	2024	Radiology	SAP, ASP	Differential diagnosis of SAP and ASP using a random forest dichotomous diagnosis model	120	97.79%–99.13% reduction in diagnosis time	C
Petrov^[23]^	2024	Radiology	Chronic subdural hemorrhage	Segmentation and volume measurement of chronic subdural hematoma on CT	21	75%, 75.22%, 83.06%, 92.16%, and 92.53% reduction in diagnosis time	A
Sarkar^[24]^	2024	Radiology	Spleen injury	Automated classification of spleen injury severity based on AAST score	76	66.67% and 88% reduction in diagnosis time	A
Wang^[25]^	2019	Radiology	Pulmonary nodules	Detection of pulmonary nodules on CT	1965	58.98% reduction in diagnosis time	A
Watkins^[26]^	2022	Radiology	Systemic cancer	Generation of 21 unique OARs and 4 PTVs from the whole body	467	75% reduction in diagnosis time	A
Wenderott^[27]^	2024	Radiology	Prostate Cancer	Prostate MRI interpretation and diagnostic reporting	91	10.48% increase in diagnosis time	A
Zeleznik^[28]^	2021	Radiology	Breast cancer	Volumetric heart segmentation on chest CT	1306	50% reduction in diagnosis time	A
Zhang^[29]^	2023	Radiology	Breast cancer	Automatic segmentation of breast tumors by capturing dynamic changes in multi-phase DCE-MRI	5627	95.30% reduction in diagnosis time	A
Zhang^[30]^	2022	Radiology	Esophageal cancer	Analysis of barium esophagram	17,797	54.6% reduction in diagnosis time	A
Yang^[31]^	2021	Pathology	Gastric cancer	Identification of cancer lesions	57	AI model 1: 98.85% reduction in diagnosis time AI model 2: 99.43% reduction in diagnosis time	A
Eloy^[32]^	2023	Pathology	Prostate cancer	Detection, grading, and quantification of prostate cancer	105	21.94% reduction in diagnosis time	A
Huang^[33]^	2021	Pathology	Prostate cancer	Detection, grading, and quantification of prostate cancer	1000	>75% reduction in diagnosis time‡	A
Da Silva^[34]^	2021	Pathology	Prostate cancer	Detection of prostate cancer	600	65.5% reduction in diagnosis time	B
Wu^[35]^	2022	Pathology	Non-small cell lung cancer	Assessment of the tumor proportion score of PD-L1 expression	40	51.58% reduction in diagnosis time	A
Steiner^[36]^	2020	Pathology	Prostate cancer	Gleason grading of prostate biopsies	240	13.5% reduction in diagnosis time	A
Oh^[37]^	2024	Gastroenterology	Small bowl lesion	Removal of poorly visualized image before reading CE	90	35.6% reduction in diagnosis time	B
Ruan^[38]^	2022	Gastroenterology	Ulcerative colitis and Crohn disease	Identification between ulcerative colitis and Crohn disease in endoscopy image	4886	Trainee VS AI → 99.74% reduction in diagnosis time Competent VS AI → 99.74% reduction in diagnosis time	C
Park^[39]^	2020	Gastroenterology	Small bowl lesion	Assistance in lesion detection by physicians using CE	20	50.37% reduction in diagnosis time	A
Zhang^[40]^	2024	Gastroenterology	Small bowel lesion	Objective detection of small bowel lesions in CE	37,287	99.17% reduction in diagnosis time	A
Salama^[41]^	2022	Internal medicine (hematology)	Chronic lymphocytic leukemia	Evaluation of the efficiency of detecting microresidual disease in CLL patients	34	98.67% reduction in diagnosis time	A
Xing^[42]^	2023	Internal medicine (hematology)	Morphological identification of peripheral leukocytes	Preclassification to verify that classification time is reduced	102	79.63% reduction in diagnosis time	A
Katz^[43]^	2021	Internal medicine (hematology)	Abnormalities of blood cellular components	Determining the shape of platelets, WBC, RBC on PBS	645	62.7% reduction in diagnosis time	A
Lv^[44]^	2022	Internal medicine (cardiovascular medicine)	Aortopathies	Estimation of time-averaged wall shear stress	154	99.93% reduction in diagnosis time	C
Gu^[45]^	2024	Internal medicine (cardiovascular medicine)	Stroke	Automatic assessment of stroke severity (NIHSS score) and classification of disease severity	386	98.42% reduction in diagnosis time	C
Yan^[46]^	2023	Ophthalmology	Normal/abnormal cornea	Recognition of corneal layers in IVCM images and their classification as normal or abnormal	580	99.72% reduction in diagnosis time	C
Yang^[47]^	2022	Ophthalmology	Diabetic retinopathy	Detection of diabetic retinopathy	962	37.32% reduction in diagnosis time	C
Chiu^[48]^	2022	urology	Urinary tract infection	Measurement of the time required to process urine samples using AI	1519	59.52% reduction in diagnosis time	C
Zhao^[49]^	2020	Nuclear medicine	Bone metastasis	Diagnosis of bone metastasis in bone scintigraphy	400	99.88% reduction in diagnosis time	C
Lu^[50]^	2021	Neurological surgery	Brain tumor	Lesion detection and segmentation on brain tumor SRS	10	30.08% reduction in diagnosis time	A

AAST = American association for the surgery of trauma, AI = artificial intelligence, ASP = Aspergillus pneumonia, BA = bone age, CE = capsule endoscopy, CLL = chronic lymphocytic leukemia, CMR = cardiovascular magnetic resonance, CT = computed tomography, CXR = chest radiography, DBT = digital breast tomosynthesis, DCE-MRI = dynamic contrast-enhanced magnetic resonance imaging, IVCM = in vivo confocal microscopy, MRI = magnetic resonance imaging, NIHSS = national institutes of health stroke scale, OAR = organs at risk, PBS = peripheral blood smear, PD-L1 = programmed death-ligand 1, PTV = planning target volumes, qXR = AI-based chest X-ray screening tool, RBC = red blood cell, SAP = Staphylococcus aureus pneumonia, SRS = stereotactic radiosurgery, WBC = white blood cell.

* All studies in the table demonstrated statistically comparable or higher accuracy and sensitivity during the validation phase and in performance comparisons with clinicians.

† Provision of supporting material for clinicians’ decision-making (A), reduction in the volume of data requiring review during diagnosis (B), and independent diagnosis by AI (C).

‡ Pathologists typically required 4 to 6 minutes per slide for grading, quantification, and diagnosis, but with the AI-assisted method, the time was reduced to less than 1 minute per slide.

**Table 2:** AI Applications and Data volume Reduction Across Medical Specialties*

Lead author	Year	Specialty	Disease	Outcome	Sample size	Result	ABCD classification†
Shoshan^[51]^	2022	Radiology	Breast cancer	Diagnosis of breast cancer in DBT	5182	39.6% reduction in workload	D
Lancaster^[52]^	2022	Radiology	Lung cancer	Detection of pulmonary nodules	283	77.4–86.7% reduction in workload	D
Rodriguez-Ruiz^[53]^	2019	Radiology	Breast cancer	Breast cancer detection in DM and DBT	2654	17% reduction in workload	D
Seker^[54]^	2024	Pathology	Breast cancer	Early detection and interval cancer detection in breast cancer screening	5136	69.5% reduction in workload	D
Vermorgen^[55]^	2024	Pathology	Endometrial cancer	Classification of normal, abnormal, and malignant endometrial tissue	91	51.03%–72.9% reduction in workload	D
Peltola^[56]^	2023	Neurology	Epilepsy	Detection of epochs and classification of seizure types	40	86% reduction in workload	D

AI = artificial intelligence, DBT = digital breast tomosynthesis, DM = digital mammography.

* All studies in the table demonstrated statistically comparable or higher accuracy and sensitivity during the validation phase and in performance comparisons with clinicians.

† Volume reduction of data requiring review without time measurement (D).

## 4. Discussion

Of the 51 studies reviewed based on the 2 quantifying factors, 44 showed a significant reduction in diagnostic time with AI, 6 also showed a significant reduction in data volume, and only 1 study showed an increase in diagnostic time. Our results have shown that AI is helpful in increasing productivity, precision, and task management in various sectors and has brought a complete revolution in the diagnosis of diseases. Most AI tools provide annotated data, predictive models, and preprocessed results, thereby reducing the cognitive load on medical personnel. The main advantages of AI are that it can automate time- and resource-intensive tasks, such as morphological analysis, lesion detection, and image segmentation, allowing physicians to concentrate their efforts on higher-order decision-making. AI helps improve workflow efficiency through dramatic cuts in diagnosis time, often by over 90% in tasks related to lesion detection and bone metastasis analysis. Furthermore, AI can use pattern recognition to identify subtle abnormalities that humans may not detect. This enhances the diagnostic accuracy. In a few areas, such as hematology and cardiology, AI has standardized diagnosis by providing consistent and reproducible results that are less susceptible to individual clinician experience.

The integration of AI into medical diagnosis holds promise, with increasing accuracy reported to date, thus possibly reducing the workload and filling gaps in resources developed within different clinical contexts as a building block for both efficient and effective healthcare.

The specific characteristics and differences in AI applications across various medical disciplines are described below.

### 4.1. Radiology

AI has been researched and clinically utilized in radiology since the early days of AI deployment, and its efficiency has been proven to be the best. In this study, AI had a revolutionary impact on radiology, significantly reducing the workload and improving the diagnostic efficiency. For example, when diagnosing breast lesions in contrast-enhanced mammography using AI, the processing time is shortened by up to 99.67% compared to the traditional method.^[6]^

In the field of radiology, AI is mainly used for image interpretation and lesion detection and analyzes data such as computed tomography (CT), magnetic resonance imaging, and X-rays. There were many cases of use for all 4 categories (A, B, C, and D). Among them, the proportion of cases classified as C was particularly high compared with that in other departments. Examples of A include, in the diagnosis of lung disease, AI labeling the normal structure of chest CT, detecting and segmenting lung lesions, and then measuring the number, location, and size, as well as evaluating the lung parenchyma and measuring cardiac volume and coronary artery calcium volume.^[7]^ Simply put, AI automatically analyzed the findings on the heart, lungs, and musculoskeletal system to provide datasets to the clinician. Subsequently, the clinician was able to shorten the time by 22.1% by reading this AI analysis result. Categories B and D have AI models for digital breast tomography, which are increasingly used for breast cancer screening worldwide. In a study by Raya-Povedano et al,^[8]^ AI helped clinicians with digital breast tomography-based screening strategies, reducing data volume by up to 70% and time by 72.2%. In addition, Shoshan et al^[51]^ proposed an AI model to detect cancer-free screenings that could have been dismissed without consulting a clinician and reduced the data volume by 39.6%, while the time change was not given as an exact figure. Unlike previous studies, Li et al^[9]^ showed that the deep learning-based automatic fresh rib fracture detection and positioning system reduced the time it took to detect fresh rib fractures by 95% when diagnosed independently compared to clinicians, which is an example of category C.

There were more cases classified as category C, an independent diagnosis of AI, compared to other departments, because the data were digitized from the beginning of diagnosis. The fact that the interpretation of radiology images mostly follows a standardized protocol must also be considered; major radiology tools, such as CT scans and X-ray images, have a distinct resolution and format, making it easy for AI to consistently process the data and recognize abnormal patterns. Radiology is a field that performs quantitative analysis using clear visual data. AI is advantageous for repetitive and quantifiable tasks, such as lesion detection, and therefore, shows high accuracy in analyzing medical images. In other words, AI can sufficiently demonstrate the advanced pattern recognition ability necessary to make independent diagnoses. These developments show that the usefulness of AI in radiology is wide-ranging, from assisting clinicians to performing fully automated diagnosis.

However, there were some studies with conspicuous results. First, an AI that diagnoses intracranial aneurysms showed higher sensitivity and faster diagnosis time than clinicians, but it showed significantly lower specificity when compared with clinicians.^[10]^ This was attributed to small positive samples due to the low prevalence of intracranial aneurysms.

Second, AI detecting pneumonia lesions in COVID-19 patients showed higher sensitivity and faster diagnosis time than clinicians, but the specificity was significantly lower.^[11]^ This was attributed to the algorithm’s recognition of metallic or respiratory-labeled artifacts or fibrosis as a lesion of COVID-19. In addition, when AI was used as a tool to support clinicians, it showed higher sensitivity than when clinicians diagnosed alone while maintaining high specificity, and the diagnosis time was also reduced.

Third, an AI that detects and classifies pulmonary nodules was compared with 5 experienced clinicians. AI performed better in negative misclassifications than clinicians.^[52]^ However, it showed more positive misclassifications than clinicians, which was attributed to overestimating the size of nodules attached to blood vessels or the pleura.

Fourth, a study also reported that case reading times were not significantly reduced when AI was implemented in actual diagnostic workflows.^[27]^ Rather, reading time was increased by 10.48%, which appears to be due to the fact that it takes 10 to 20 minutes to upload images to the platform. This study shows that further research and optimization of diagnostic workflows are needed to effectively integrate AI into clinical practice environments and increase work efficiency.

### 4.2. Pathology

AI has demonstrated significant benefits in pathology, particularly in cancer diagnosis. Using AI models, gastric cancer lesion identification reduced the diagnostic time by up to 99.43%.^[31]^ Similarly, AI-assisted Gleason grading of prostate biopsies decreased diagnostic time by 21.94% while reducing requests for additional immunohistochemical (IHC) studies and second opinions by 20.72% and 39.21%, respectively.^[32]^ The reduction in IHC testing and second-opinion requests has significant implications for reducing the workload of clinicians during the diagnostic process. This includes minimizing the tasks associated with conducting additional IHC tests and reviewing new IHC test results or second opinions.

The results of previous studies highlight the significant potential of AI to improve clinicians’ diagnostic efficiency by providing supporting material for clinician decision-making (category A in our study). This includes the study by Huang et al,^[33]^ which presented AI-enabled segmentation and labeling of images to reduce diagnostic time by at least 75%, identifying each cancer gland or epithelial patch with its corresponding Gleason pattern on the tissue.

In 2021, Paige Prostate, an AI-based system for automatic prostate cancer detection, is remarkable in that it directly demonstrated that AI can reduce the number of slides that need to be read during the diagnostic assistance process, thereby shortening diagnosis time.^[34]^ The AI developed in this study reduced the number of slides that a clinician must review from 579 to 200, which shortened the average diagnosis time from 15.76 hours to approximately 6.77 hours, a 65.5% reduction. These results are based on actual diagnostic data compared to the consensus diagnosis of clinicians and were achieved while maintaining specificity, high sensitivity, and negative predictive value. This demonstrates the AI potential of category B in our study to safely reduce clinicians’ data volume without compromising diagnostic quality through filtering using AI.

Pathology presents more challenges than radiology in the application of AI in the diagnostic process. While radiological images are already standardized in digital formats to facilitate AI model training, pathology requires digitizing tissue samples on glass slides, a process complicated by factors such as resolution, color variability, and the inherent diversity of tissue structures.

For example, while objective results showed that AI-assisted clinicians had better diagnostic agreement, some diagnostic discrepancies were related to low-quality IHC staining during the preparation and digitization of tissue.^[35]^ This study also pointed out that the nature of pathological data makes data collection very difficult and often leads to relatively small datasets. The diagnostic skills of AI in pathology will be significantly enhanced by overcoming these limitations, thus enabling impactful application in this field.

### 4.3. Gastroenterology

In gastroenterology, 3 of the 4 studies introduced AI models related to capsule endoscopy (CE). In the case of CE, clinicians are required to review many images taken at the same anatomical location, leading to a significant workload. Therefore, the potential demand for shortening the CE diagnosis time results in a large proportion. Examples of categories A and B include a study that reduced the diagnosis time for AI-assisted reviewers by 50.37% and a study that used AI to remove poorly visualized images from small bowel CE, resulting in a 35.6% reduction in diagnosis time.^[37,39]^

Because the lesion data in gastroenterology are relatively atypical, and the size and location of the lesions are varied, the AI must be trained and validated with images that are similar to those obtained from actual clinical situations. However, in the present study, AI was unable to detect uncommon lesions that were not included in the training samples.^[39]^

In addition, clinicians should observe the colon in real time and focus on its anatomy, lesion size and count, stenoses, and unaffected areas. One of the studies reviewed belongs to category C, which demonstrated an AI differentiating Crohn disease from ulcerative colitis nearly instantaneously.^[38]^ However, another study developed an AI that detects lesions with only images after endoscopy has been completed.^[40]^ Therefore, large-scale studies are needed to confirm the performance of AI and evaluate its real-time diagnosis in the future.

### 4.4. Neurology

The contributions of AI to neurology, though limited in number, are impactful. For example, in epilepsy monitoring, AI reduced the video run time required for clinician review by 86.2% and detected seizures with 100% sensitivity, which is classified as category D.^[56]^ However, although this system showed significant agreement in seizure classification when compared with the gold standard using Gwet agreement coefficient, it did not support myoclonic seizures, tonic seizures, or epilepsy. There were limitations in accurately detecting individual motor seizures such as convulsions. Future research should address false-positive rates and explore the applicability of AI to broader neurological conditions.

### 4.5. Hematology

In hematology, AI is mainly used as auxiliary data to determine the shape of the blood cells. Morphological identification of blood cells is somewhat subjective and can lead to different results depending on the experience of a skilled clinician. In hematology, we have seen a lot of category A studies that help AI automate tasks such as evaluating peripheral blood smears and detecting chronic lymphocytic leukemia, allowing them to perform diagnoses faster and more accurately than clinicians can alone. For example, AI detected microscopic residual disease in leukemia patients, resulting in a time reduction of 98.67%,^[41]^ and peripheral leukocyte sorting was also reduced by 79.63%.^[42]^ Peripheral blood analysis showed a 62.7% time-saving.^[43]^ These results suggest the possibility of AI standardizing hematologic diagnosis; however, more research is needed to ensure consistency across laboratories.

### 4.6. Cardiovascular medicine

AI has fairly enhanced the diagnostic efficiency in the cardiovascular medicine field, and this was particularly remarkable in tasks requiring complicated calculations. For instance, AI-driven estimation of time-averaged wall shear stress in aortopathies achieved a 99.93% reduction in time,^[44]^ and AI in stroke severity assessment similarly demonstrated substantial time reduction.^[45]^ These results highlight the potential of AI to fall into category C because it independently performs time-consuming diagnostic tasks.

### 4.7. Ophthalmology

In ophthalmology, AI has been effectively applied to accelerate diagnostics. For instance, diabetic retinopathy detection using AI reduced diagnostic time by 37.32%, and an AI model that classified corneal abnormalities reduced time by 99.72% while achieving expert-comparable accuracy.^[46,47]^ They are classified as category C, which highlights the potential of AI for use in clinical education.

### 4.8. Urology

In urology, when AI was used alone, the processing time for urinary tract sample analysis was reduced by 59.52% compared with the clinician’s diagnosis, which is category C.^[48]^ Urine culture is a major test in microbiology laboratories and requires significant effort. The introduction of automation systems using AI can improve accuracy and efficiency. In the future, it is thought that AI can be integrated into the urological diagnostic process to quickly prescribe appropriate antibiotics for patient care.

### 4.9. Nuclear medicine

In nuclear medicine, AI can independently diagnose bone metastases through bone scintigraphy, resulting in a 99.88% reduction in time, and is classified as category C.^[49]^ After AI consultation, however, the accuracy and sensitivity increased and the false-negative rate dropped, although the time cost of clinicians was prolonged. This demonstrates the transformative potential of AI in automating complex imaging tasks, although further studies are needed to validate these findings across diverse clinical contexts.

### 4.10. Neurological surgery

In neurosurgery, the manual segmentation process is time-consuming and can vary considerably between clinicians. In brain tumor diagnosis, AI-assisted segmentation reduced the time by 30.08%, highlighting its potential in neurological surgery.^[50]^ This study was classified as category A, and the false-positive rate was higher when ABS was used alone than by clinicians; however, when ABS and clinicians collaborated, it showed higher accuracy, lesion detection sensitivity, and contouring accuracy than when ABS or clinicians split tumors independently. This suggests that collaboration between AI and clinicians can result in better diagnostic performance than when using either approach alone. Further validation is required to ensure consistent results across different surgical scenarios.

### 4.11. Addressing global healthcare challenges

Our analysis now incorporates studies from various regions to minimize regional bias and addresses global challenges, such as workforce shortages and cost-effective diagnostics. Additionally, we framed our findings in the context of international healthcare policy and workforce planning, ensuring broader applicability and resonance with an international audience.

### 4.12. Limitation of study

Our study has several limitations. We only used PubMed as the article search database. This led to excluding articles accessible through other databases, potentially narrowing the scope of our analysis. Additionally, we excluded non-English papers and studies from local journals, thus limiting the diversity of our analysis.

This study focused only on improving the efficiency of the process of disease diagnosis, which resulted in several limitations. AI systems are not only able to diagnose disease but can also be applied across healthcare, including treatment decisions, patient classification, prognosis prediction, and the development of efficient prevention methods. Therefore, the influence of the AI identified in this study, which focuses solely on the diagnostic process, might be underestimated. Further studies are needed to understand the trend and degree of development of AI systems that can be used in processes other than diagnosis. Moreover, we defined the improvement in diagnostic efficiency as time and data volume reduction in the diagnostic process. Factors that may also affect the diagnostic efficiency of clinicians, such as increased diagnostic accuracy of auxiliary AI and shortened diagnostic procedures, were not considered in our study. Thus, future studies should consider various approaches that can effectively reduce the workload of clinicians.

In addition, there were some limitations to the studies we reviewed. Many studies have shown limitations regarding research design or data interpretation. For instance, one of the reviewed studies did not include the time required for AI to enter information into the diagnostic process.^[38]^ With the possibility of similar limitations in other studies we reviewed, the result might have been biased, potentially concluding that AI performance is more powerful than it truly is. Additionally, another study concluded that the time cost of diagnosis increased when applied to a real-world clinical workflow owing to the time for uploading data to the AI system.^[27]^ Moreover, the number of clinicians was very small compared to that in the AI models, which could not adequately account for the variability in their performance. In the comparison between clinicians and AI models, clinician groups were relatively small, with a median size of 4 (interquartile range: 3–7.5; range: 1–32; 4 studies in which the number of clinician comparators was not specified were excluded from the calculation of the median value).^[22,26,54,56]^ Therefore, future investigations should involve a larger cohort of healthcare professionals to ensure robust comparisons with AI in terms of their efficiency. Above all, many of the studies we reviewed compared the results of clinicians with those of independent AI diagnosis models, which were difficult to apply in real workflows due to ethical and legal constraints.^[57]^ Although AI models have shown remarkable potential to enhance diagnostic efficiency, with studies reporting over 99% reductions in time cost for diagnosis, such as bone metastasis diagnosis, many restrictions remain regarding the independent application of AI in real-world medical settings.^[49]^

Finally, AI itself has some limitations. Samples for rare diseases are often scarce, which causes AI models to face significant challenges in training. Several studies have reported difficulties in diagnosing rare diseases due to insufficient training data, which is a common limitation in AI models that rely on large, diverse datasets for effective learning.^[12]^ Hence, future studies should focus on addressing problems associated with diagnosing rare diseases.

In addition, several studies indicated that AI applications effectively reduced workload in medical specialties. These findings are summarized in Tables 1 and 2.^[13–21,23–25,28–30,36,53,55]^

## 5. Conclusions

AI has changed the paradigm of medical diagnosis by increasing the precision, speed, and harmonization across a wide range of specialized areas. By automating repetitive tasks such as lesion detection, image segmentation, and morphological analysis, AI reduced the diagnosis time by over 90% in some studies and data volume by over 85%, thus greatly improving workflow efficiencies. It also delivers reliable and repeatable results with a significant reduction in volatility owing to differences in clinician expertise. Although the most apparent potential of AI lies in radiology and pathology, challenges remain, such as nonstandardized data formats in pathology and ethical considerations with real-world applications. Overcoming these issues and ensuring further optimization of how AI is integrated into clinical workflows will be crucial to the full realization of this transformative potential. Future research and development should focus on broadening the scope of applications while ensuring consistency, reliability, and adaptability to meet diverse healthcare needs. Furthermore, integrating AI into healthcare workforce planning is essential, fostering seamless collaboration between technology and clinicians to enhance its effectiveness in patient care.

## Author contributions

**Conceptualization:** Jinseo Jeong, Seok-Ju Yoo.

**Data curation:** Jinseo Jeong, Sohyun Kim.

**Formal analysis:** Jinseo Jeong, Seok-Ju Yoo.

**Methodology:** Jinseo Jeong, Daye Hwang, Seok-Ju Yoo.

**Writing – original draft:** Jinseo Jeong, Daye Hwang, Dongseop Kim, Jeongwon Choi, Yeongkyo Kwon, Pyeongro Yi, Jisoo Jeong.

**Investigation:** Sohyun Kim, Lian Pan, Daye Hwang, Dongseop Kim, Jeongwon Choi, Yeongkyo Kwon, Pyeongro Yi, Jisoo Jeong.

**Resources:** Sohyun Kim, Lian Pan.

**Validation:** Lian Pan.

**Writing – review & editing:** Dongseop Kim, Jeongwon Choi, Yeongkyo Kwon, Pyeongro Yi, Jisoo Jeong.

**Funding acquisition:** Seok-Ju Yoo.

**Supervision:** Seok-Ju Yoo.
